# Preclinical models for studying immune responses to traumatic injury

**DOI:** 10.1111/imm.13272

**Published:** 2021-01-05

**Authors:** Jessica Katy Skelton, Robert Purcell

**Affiliations:** ^1^ CBR Division Defence Science and Technology Laboratory Salisbury UK

**Keywords:** immune response, preclinical models, trauma

## Abstract

Traumatic injury initiates a large and complex immune response in the minutes after the initial insult, comprising of simultaneous pro‐ and anti‐inflammatory responses. In patients that survive the initial injury, these immune responses are believed to contribute towards complications such as the development of sepsis and multiple organ dysfunction syndrome. These post‐traumatic complications affect a significant proportion of patients and are a major contributing factor for poor outcomes and an increased burden on healthcare systems. Therefore, understanding the immune responses to trauma is crucial for improving patient outcomes through the development of novel therapeutics and refining resuscitation strategies. In order to do this, preclinical animal models must mimic human immune responses as much as possible, and as such, we need to understand the constraints of each species in the context of trauma. A number of species have been used in this field; however, these models are limited by their genetic background and their capacity for recapitulating human immune function. This review provides a brief overview of the immune response in critically injured human patients and discusses the most commonly used species for modelling trauma, focusing on how their immune response to serious injury and haemorrhage compares to that of humans.

## Graphical abstract

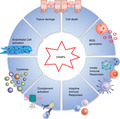

**Abbreviations**
ATP: adenosine triphosphate
CARS: compensatory anti‐inflammatory response syndrome
CCR5: C‐C chemokine receptor type 5
CD: cluster of differentiation
DAMPs: damage‐associated molecular patterns
DNA: deoxyribonucleic acid
G‐CSF: granulocyte‐colony stimulating factor
HMGB1: high‐mobility group box protein 1
IFN: interferon
Ig: immunoglobulin
IL: interleukin
IRF: interferon regulatory factor
LPS: lipopolysaccharide
Ly46: lymphocyte antigen 46
NF‐κB: nuclear factor kappa B
NHP: non‐human primates
NK: natural killer
MHC: major histocompatibility complex
MODS: multiple organ dysfunction syndrome
Mx1: myxovirus resistance‐1
NETs: neutrophil extracellular traps
PRR: pattern recognition receptor
ROS: reactive oxygen species
SIRS: systemic inflammatory response syndrome
TBI: traumatic brain injury
TBV: total blood volume
TNFα: tumour necrosis factor α
TLR: toll‐like receptor

## INTRODUCTION

Traumatic injury is one of the leading causes of death worldwide, accounting for around 9% of the total number of deaths globally each year.^1^ Improvements in the management and treatment of haemorrhage in critically injured patients have reduced mortality in both civilian^2^ and military^3^ settings. However, in patients that survive the initial injury, a significant number will go onto to develop sepsis or multiple organ dysfunction syndrome (MODS) in the subsequent days and weeks. These complications are associated with a poor prognosis^2^, ^4^, ^5^ and are believed to be a result of immune dysfunction.^6^ Even in patients that survive, developing these complications is a significant burden on healthcare systems due to prolonged hospitalization and increased resource requirement.^5^ It is now known that traumatic injury initiates a complex and dynamic immune response within minutes of the initial injury^6^, ^7^; these responses are not a result of any infection, but are a direct response to haemorrhage and tissue damage.^7^ Typically, this ‘sterile inflammation’ is due to the release of damage‐associated molecular patterns (DAMPs) from damaged and necrotic cells that result in immune cell^8^ and complement^9^ activation, and cytokine release.^10^ Severe injury can lead to the development of systemic inflammatory response syndrome (SIRS) and a compensatory anti‐inflammatory response syndrome (CARS). Although traditionally believed to be distinct and separate processes that occurred sequentially, recent evidence suggests that these actually occur concurrently, contributing to the increased risk of sepsis and the development of MODS.^7^ Thus, targeting the immune system in critically injured patients may be a viable strategy for improving patient outcomes and mortality rates, an approach that is currently the subject of clinical trials (e.g. TOP‐ART study to investigate the use of artesunate to prevent organ dysfunction). It is therefore essential to have preclinical models that replicate human immune responses to trauma in order to enhance mechanistic understanding and for the development of novel therapeutic strategies. This review will firstly briefly summarize what is currently known about the immune response to traumatic injury in humans, and then will discuss the advantages and pitfalls of various preclinical models that are commonly used to study these responses. As the cause, type and severity of trauma in humans is heterogeneous, for the purposes of this review, ‘traumatic injury’ and ‘trauma’ will be used interchangeably as general terms to describe severe injury and haemorrhage; specific injuries will be referred to where appropriate.

## THE IMMUNE RESPONSE TO TRAUMATIC INJURY

**FIGURE 1:**
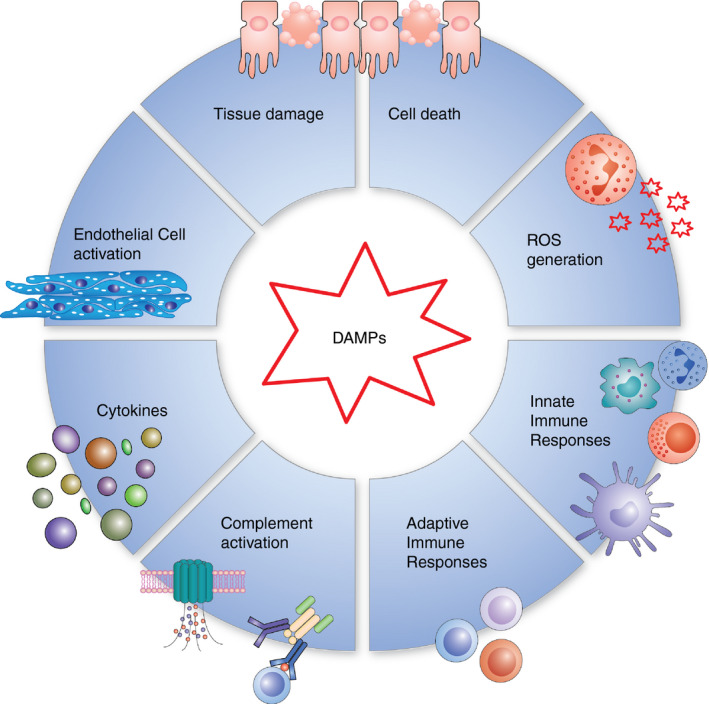
The role of DAMPs in traumatic injury. DAMPs released following traumatic injury initiate systemic inflammatory responses, such as the release of ROS and cytokines and the activation of the endothelium and immune cells. Together, this extensive inflammatory response contributes to further tissue damage and cell death

Traumatic injury initiates concurrent inflammatory (i.e. SIRS) and anti‐inflammatory responses (i.e. CARS),^11^, ^12^, ^13^ driven by the release of DAMPs from damaged and necrotic cells following tissue injury and haemorrhagic shock (Figure 1). DAMPs, via the activation of pattern recognition receptors (PRRs), activate complement^9^ and immune cells,^8^, ^14^ instigating the release of cytokines that initiate and propagate the systemic inflammatory response.^7^, ^15^, ^16^ There is also emerging evidence, suggesting that DAMP levels are associated with immune suppression following traumatic injury^13^ and surgery.^17^ Although the mechanistic basis is not entirely clear, there have been suggestions that ‘suppressive DAMPs’ are released by activated leucocytes and may contribute to a prolonged period of CARS and immune suppression.^15^ DAMPs that have shown to be increased in the circulation following trauma include high‐mobility group box protein 1 (HMGB1),^18^ heat shock proteins^13^ and mitochondrial and nuclear DNA^19^; the role(s) of DAMPs in trauma has been extensively reviewed elsewhere.^15^, ^16^ The most widely studied DAMP following trauma, HMGB1, is significantly elevated in the plasma of trauma patients within an hour of injury,^14^, ^15^ with levels correlating with injury severity^18^ and the development of sepsis and MODS.^20^ Studies *in vitro* have demonstrated that HMGB1 has a wide range of pro‐inflammatory actions on immune cells such as promoting cytokine secretion,^14^ reactive oxygen species (ROS) production^21^ and chemotaxis.^22^ Moreover, HMGB1 has direct effects on the vascular endothelium, such as up‐regulating adhesion molecule expression and cytokine release,^23^ promoting neutrophil adhesion^23^ and increasing endothelial permeability.^24^ Endothelial activation and glycocalyx shedding^25^, ^26^, ^27^ are early manifestations of traumatic injury and are believed to be caused by DAMPs, catecholamines, ROS and cytokines, all of which are elevated following trauma. This ‘endotheliopathy of trauma’ is predicted to occur within minutes of the initial insult,^28^ disrupting the barrier function of the endothelium, and therefore increasing fluid leakage and leucocyte transmigration into tissues. Furthermore, hyaluronic acid^29^ and heparin sulphate^30^ fragments that are shed from the endothelial glycocalyx can serve as DAMPs, further stimulating local immune responses.

**FIGURE 2:**
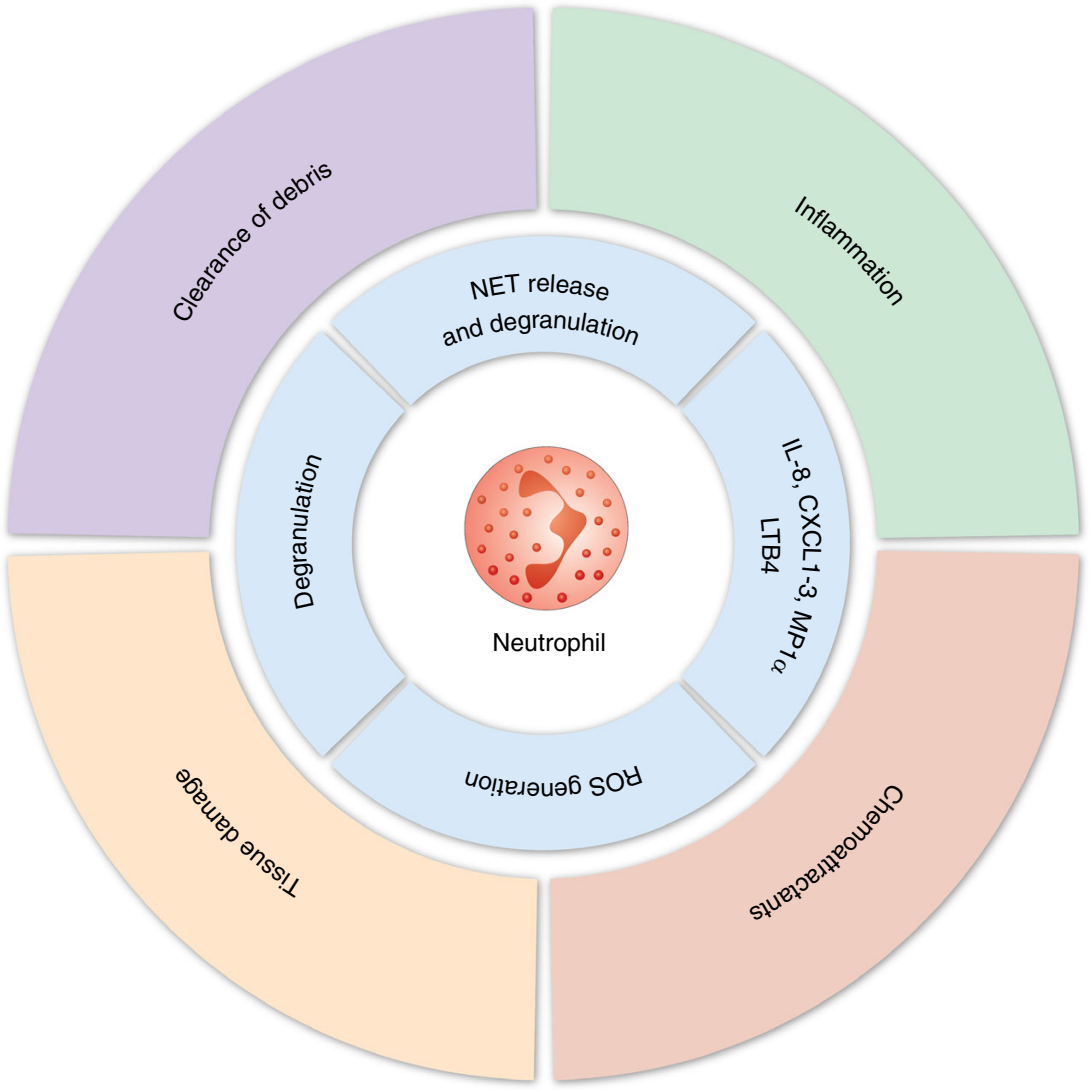
The contribution of neutrophils to the inflammatory response to injury. Following traumatic injury, elevations in circulating neutrophils are initiated by the release of chemoattractants and demargination from the intravascular pool. Additionally, neutrophils become activated, resulting in the release of proteases, NETs, ROS and cytokines. Whilst these processes can result in the clearance of tissue debris and pathogens, they can also lead to further endothelial activation, inflammation and tissue damage

**FIGURE 3:**
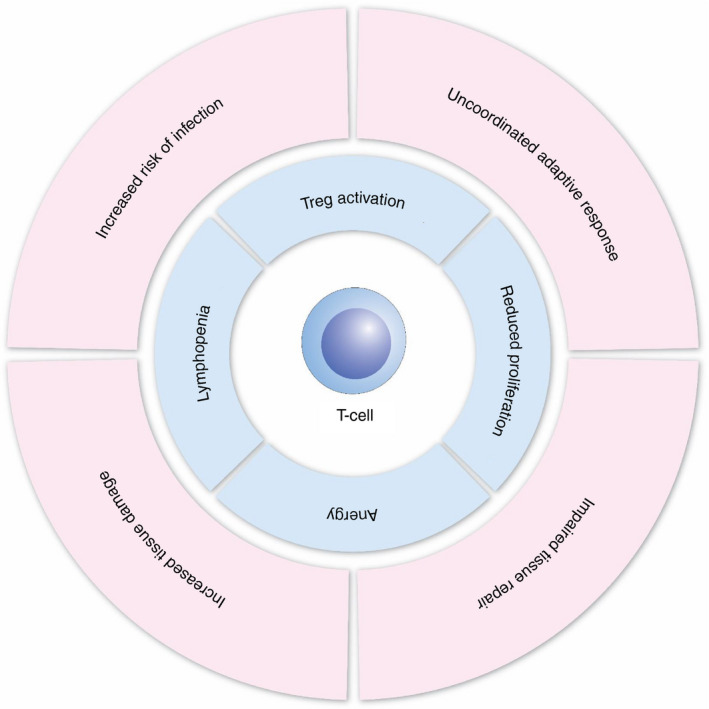
T‐cell dysregulation following traumatic injury. Although there is an initial increase in T‐cell numbers following trauma, the subsequent lymphopenia accompanied by anergy and increased immunosuppressive nature causes immune dysregulation in the hours and days that follow. As a result, T cells cannot orchestrate the adaptive immune response leaving patients more susceptible to infection, with increased tissue damage and impaired capacity for tissue repair

It is believed that a key initiating factor in the systemic inflammatory response to trauma is DAMP‐induced cytokine production.^31^ Numerous studies have shown increased concentrations of circulating pro‐inflammatory cytokines/chemokines in critically injured patients, including interleukin (IL)‐1β, IL‐6, IL‐8, granulocyte‐colony stimulating factor (G‐CSF) and tumour necrosis factor α (TNFα).^10^, ^12^, ^13^, ^32^ These changes are apparent very early after the initial injury^12^, ^33^ and persist in the hours and days that follow.^12^, ^13^, ^32^, ^33^ Simultaneously, a number of anti‐inflammatory mediators such as IL‐10, transforming growth factor‐β1 and IL‐1 receptor antagonist are also increased in the circulation.^12^, ^33^ Patients who develop MODS have increased pro‐ and anti‐inflammatory cytokine levels at hospital admission compared to those that do not develop MODS.^10^, ^32^, ^33^ Interestingly, compared to healthy controls, whole blood from trauma patients stimulated with lipopolysaccharide (LPS) shows reduced cytokine/chemokine production,^12^, ^13^ which may contribute to the increased susceptibility to infection that follows severe injury.^4^ Along with DAMPs, pro‐inflammatory cytokines serve to activate the innate immune response following injury. Together, these factors stimulate the mobilization of neutrophils from the bone marrow and their demargination from the intravascular pool.^34^ As a result, the number of circulating mature neutrophils is rapidly increased (<1 h), and they remain elevated for ~72 h.^12^, ^35^ Interestingly, the number of circulating immature granulocytes also increased in trauma patients within the same time frames,^12^, ^36^ which is likely to impact on their ability to have antimicrobial actions. Traumatic injury also initiates significant changes in neutrophil function and phenotype (Figure 2), which has been reviewed extensively elsewhere.^37^ For example, the surface expression of CD62L (reduced) and CD11b (increased), indicative of neutrophil activation, has been reported in critically injured patients.^12^ Furthermore, neutrophils isolated from trauma patients have an increased oxidative burst capacity,^38^ increased neutrophil extracellular trap (NET) production^36^ and a prolonged life span due to impaired apoptosis.^39^ However, they have a reduced phagocytic capacity^38^ and, when stimulated *ex vivo*, demonstrate a reduced capacity for integrin up‐regulation, ROS generation and NET release.^12^, ^36^ Furthermore, a neutrophil subset that suppresses T‐cell activation and proliferation^40^ is elevated in the circulation within an hour of injury, which persists for up to 72 h.^12^. These immunosuppressive neutrophils along with reduced effector functions (e.g. impaired phagocytosis and responsiveness) may contribute to the increased susceptibility to infection that is observed in trauma patients.^4^ Similarly, in the hyper‐acute (<2 h) window following injury, there is increased lymphocyte number^12^, ^35^ and activation,^32^ with the former largely being attributed to significant increases in the number of circulating natural killer (NK), CD4^+^ and CD8^+^ T‐cell populations.^12^, ^35^ However, as time progresses the number of circulating lymphocytes declines, and a pronounced lymphopenia has been observed in numerous clinical studies of trauma patients as early as 4 h following injury.^12^, ^32^, ^35^, ^41^ This reduction in total lymphocyte number is attributed to changes in T‐cell number rather than B cells,^12^, ^35^ with reductions in CD4^+^, CD8^+^, NK and γδ T‐cell populations all reported.^12^, ^42^, ^43^ Failure to reverse lymphopenia persists in patients with MODS^32^ and is associated with increased mortality.^41^ As well as reduced number, T cells isolated from trauma patients display reduced proliferation and IL‐2 production in response to activating stimuli (Figure 3).^44^ Similarly, leucocyte transcriptome analysis has demonstrated gene families associated with antigen presentation and T‐cell proliferation are less transcriptionally active following traumatic injury.^11^, ^35^ As a consequence, circulating T cells are less capable of reacting to invading pathogens and orchestrating the immune response, therefore increasing the risk of infection.

Although our understanding of the immune response(s) to traumatic injury has significantly increased, there are still many unknowns. For example, how different resuscitation fluids influence these responses, both acutely, and in the context of post‐trauma complications such as the development of sepsis and MODS. Recent evidence indicates that the volume of crystalloids administered within 24 hours of injury is associated with the development of MODS,^45^ suggesting that early intervention may be key. Blood products are considered a key part of damage‐controlled resuscitation and are believed to have a multitude of benefits to the critically injured patient (e.g. increasing tissue perfusion, replacing lost clotting factors and restoring endothelial function^46^). However, the effects of these types of resuscitation products on the immune response to trauma remain largely unknown. Due to the complicated nature of clinical studies, preclinical animal models are critical to enhance our basic understanding, as well as developing novel therapeutic strategies that target post‐trauma complications such as sepsis and MODS.

## PRECLINICAL MODELS OF TRAUMA

As described above, clinical studies have provided an opportunity to explore immune responses to traumatic injury in great detail, which has been achieved primarily through obtaining blood samples following injury. Preclinical animal models have the additional advantage of obtaining samples that are not readily available from humans, such as pre‐injury samples and invasive tissue/organ biopsies. The availability of these samples facilitates in‐depth molecular and cellular analysis (e.g. transcriptomics, flow cytometry), highlighting tissue‐specific responses that may contribute towards the development of post‐trauma complications such as MODS. Designing preclinical studies to investigate the immune response to traumatic injury poses many challenges, most notably being the suitability of the specific animal model chosen, how this model functions and its ability to recapitulate human immune responses. The main animal models used in studies of traumatic injury are rodents (particularly mice and rats), rabbits and pigs, but other species such as sheep, dogs and non‐human primates (NHPs) have also been utilized (Table 1). Each has its advantages; however, it is essential that the constraints of the species are considered when choosing an animal model to investigate these responses and that the model is applicable to the specific research question.

**TABLE 1:** Advantages and disadvantages of different species as preclinical models of trauma

	Advantages	Disadvantages	Immune cell composition (%)	Common uses in trauma research
Mice	Inexpensive Easy to handle Huge variety of genetic knock‐in/knockouts available Large cohorts Rapid breeding cycle Broad availability of biological reagents	Small TBV (relative to large animals) Difficult to manipulate Genetically the most different from humans Short life span	Neutrophils: 10%–25% Lymphocytes: 50%–90% Monocytes: 1%–4%	Drug metabolism and screening Mechanistic studies (i.e. transgenic mice) Traumatic brain injury Wound healing
Rat	Inexpensive Easy to handle Some genetic knock‐in/knockouts available Large cohorts Broad variability of biological reagents	Small TBV (relative to large animals) Short life span	Neutrophils: 4%–20% Lymphocytes: >70% Monocytes: 1%–4%	Haemorrhagic shock Wound healing Toxicology Mechanistic studies
Rabbit	Large TBV (relative to rodents) Inexpensive (relative to large animal models) Bone physiology similar to humans Superior humoral immune responses Comparable neutrophil responses to humans Long life span (relative to rodents)	Challenging to find biological reagents Genetically more similar to humans that rodents	Neutrophils: >30% Lymphocytes: 60‐70% Monocytes: 1%–5%	Fracture and traumatic bone injury Drug metabolism Haemorrhagic shock Tissue injury
Pig	Large TBV Some cross‐reactivity with human reagents Comparable lymphoid structures to humans Long life span (relative to rodents)	Complex medical equipment required Challenging to produce large cohorts Challenging to find biological reagents	Neutrophils: 20%–45% Lymphocytes: 30%–70% Monocytes: 1%–10%	Haemorrhagic shock Fluid resuscitation Tissue injury Coagulopathy
Sheep	Large TBV Bone physiology similar to humans’ long life span (relative to rodents)	Complex medical equipment required Challenging to produce large cohorts Challenging to find biological reagents	Neutrophils: 25%–30% Lymphocytes: 60%–70% Monocytes: 1%–6%	Coagulopathy Fracture and traumatic bone injury
Goat	Large TBV Bone physiology similar to humans Long life span (relative to rodents)	Complex medical equipment required Challenging to produce large cohorts Challenging to find biological reagents	Neutrophils: 25%–30% Lymphocytes: 60%–70% Monocytes: 1%–10%	Fracture and traumatic bone injury
Non‐human primates	Large TBV Immune responses Broad cross‐reactivity with human reagents Long life span (relative to rodents)	Complex medical equipment required Less ethically accepted Challenging to produce large cohorts	Neutrophils: 40%–60% Lymphocytes: 30%–50% Monocytes: 1%–4%	Haemorrhagic shock Fluid resuscitation Coagulopathy

### Mice

Murine models remain one of the most widely used animals to study many aspects of human disease, immune responses, pharmacological activity and infection kinetics.^47^ Through extensive investigations of their innate immune systems, many scientific breakthroughs have occurred using data obtained from mice (e.g. toll‐like receptor (TLR)‐4 signalling).^48^ The efficient recognition and signal transduction of foreign molecules are essential to prevent infection. In humans, this is co‐ordinated through PRR signalling that remains highly sensitive to stimulation from their corresponding ligand.^21^ However, many PRR pathways are highly species‐specific and may respond to different structures on the same ligand. Many species possess a natural increased tolerance against many TLR ligands (e.g. the median lethal dose for murine TLR4 stimulation using LPS is significantly higher than the dose that elicits the same febrile shock and cytokine responses in humans^49^). As such, there is a distinctly lower pro‐inflammatory serum cytokine response following LPS stimulation in mice than in humans (e.g. TNFα).^49^ Differences in other TLR signalling pathways have also been reported; for example, although mice express TLR8, they do not respond to the same ligands as human TLR8 receptors.^50^ As DAMPs activate PRRs to instigate the immune response to injury, differences in receptor sensitivity must be considered when designing studies to explore therapeutic approaches to target these pathways.

The composition of immune cells is distinct in mice and is variable between strains.^51^ Whilst humans display a significantly larger proportion of circulating neutrophils (50%–70%) and a lower lymphocyte percentile baseline (20%–40%), typically murine immune systems are comprised of less neutrophils (10%–25%) and a significantly increased lymphocyte percentile (50%–90%).^51^ Despite this, a comparable rapid (<6 h) immune response to trauma has been described in mice models with increased neutrophil and decreased lymphocyte numbers.^52^ Similarly, the expression of CXCR3 on NK cells is rapidly upregulated following injury, alongside increased numbers of NKT cells and γδ T cells in the peripheral circulation. In the same study, variable cell and cytokine (IFNγ, TNFα) responses in the spleen, lung and bone marrow were observed, highlighting the importance of these models in furthering our understanding of localized responses.^52^

Many immune functions significantly differ between mice and humans such as the regulation of NK cells. Human NK cells are educated through killer immunoglobulin‐like receptors during their self/non‐self‐education process, whereas murine NK cells use an alternative MHC‐I inhibitory pathway involving Ly46, which is not expressed in humans. Furthermore, the consistent functional NK cell activity exhibited throughout the human lifetime alongside the typically elevated levels of surface Fc receptor makes them distinct from murine NK cells, whereas murine NK cells display unusually high activity levels in the lung, low Fc‐receptor expression and a peaked activity in early life.^53^ That said, reductions in circulating NK numbers have been observed in a murine model of trauma and haemorrhagic shock,^52^ consistent with findings reported in humans.^12^

The differences observed between murine and human immune responses extend to significant protein expression changes and distribution. In particular, neutrophilic granule content in mice contains significantly lower myeloperoxidase, lysozyme, β‐glucuronidase, alkaline phosphatase and lysozyme enzymatic activity than that of humans.^54^ Murine defensins are found in paneth cells in the gastrointestinal tract and are not expressed in neutrophils, which is in stark contrast to the 4 defensins that comprise 50%–70% of azurophilic granule content in human neutrophils.^54^ Additionally, distinguishing M1 and M2 polarized macrophages is distinct in mice and humans, with macrophage phenotypes defined using markers that are not expressed in the other species (e.g. matrix metalloproteinase‐1 is not expressed in murine M1/M2 macrophages and platelet‐derived growth factor‐C is not expressed in human M1/M2 macrophages).^54^ Ultimately, there are many significant variations in the immune responses observed in mice to that of humans. Due to excessive inbreeding, many strains of laboratory mice have intrinsic genetic defects that alter their capacity for a holistic recapitulation of human immune responses (e.g. removal of the *Mx1* gene).^54^

Mice have been used to model different types of injury, most commonly haemorrhage, traumatic brain injury (TBI) and bone fracture,^47^ with many studies focusing on immune responses and possible interventions to improve prognosis.^55^ These models elicit many of the early responses as humans, such as endothelial barrier disruption,^56^ and elevations in circulating levels of DAMPs and pro‐inflammatory cytokines.^47^ Fixed‐pressure haemorrhage in mice alters dendritic cell function with depressed production of IFNγ, MHC‐II and IL‐12.^57^ Additionally, combinatorial models have also been produced in mice; for example, splenic macrophages demonstrate increased TNFα and IL‐6 levels following 40% total blood volume (TBV) fixed‐volume haemorrhage and femur fracture.^58^ Similarly, alveolar macrophages express elevated TNFα and macrophage chemotactic protein‐1 following ischaemia/reperfusion lung injury.^59^

The advantages of using mice models besides their small size are their accessibility, lack of expense and availability of biological reagents (e.g. antibodies), and they are generally regarded as more ethically acceptable as an animal model. Additionally, the rapid breeding cycles and easy manipulations have allowed the generation of many desirable genetic knockout strains.^60^ However, the small anatomy of these animals makes performing experiments more challenging, and in many respects, they do not fully recapitulate the complex immune networks and responses observed in humans.^61^ Despite their differences, 80% of murine genome is same as humans and many of these similarities have furthered our understanding of immune responses, which makes them an invaluable tool.^47^

### Rat

Using alternative rodent models is a common preference, as rats boast all of the same advantages (reagent availability, low cost and ethical implications) as murine models, but are larger in size and possess a larger TBV that can make performing many procedures less challenging (e.g. shock injury).^48^ The rat genome is composed of approximately 90% orthologs of human genes.^62^ Unlike the mice, many immune‐related genes that are shared between rats and humans have evolved through genetic expansion, and therefore, many of the principles of human immunology apply to these models. Many of these gene families are not present in the mice, and there is only a 30% alignment between the rat and mice genomes.^63^ Despite this, the elevated lymphocyte (>70%) and reduced neutrophil (4%–20%) percentiles mean that rat immune composition is more similar to mice than humans.^64^ However, this varies between different laboratory rat strains.^65^

Like mice, rats are comparatively easy to genetically manipulate, but possess more physiological similarities with humans than mice. This often makes them the preferred choice for many preclinical investigations (e.g. pharmacokinetics, toxicology).^66^ Thus, there have been many rat models of traumatic injury developed that include response to wound injury,^67^ bone fracture and repair,^68^ traumatic haemorrhage^47^ and blast injury of the lung.^69^ Blunt chest trauma induces comparable monocyte migration to the lung in rats as in humans.^59^ In contrast, although rat neutrophils express the leukotriene B_4_ receptor, they have a reduced capacity to migrate towards this chemoattractant than human neutrophils.^70^ Additionally, fixed‐pressure haemorrhage and trauma laparotomy results in increased permeability in the gut affecting neutrophil priming through mesenteric lymph nodes.^71^ Rat alveolar macrophages have been demonstrated to display increased phagocytic capacity and reduced IL‐10 production following haemorrhagic shock, responses that have also been described in human patients of haemorrhagic shock.^59^, ^72^

There are several limitations that must be considered with rat models. Vitamin D receptors are crucial components of innate immune signalling in humans and are fundamental to antigen‐presenting cells and lymphocytes. Rat vitamin D receptors however do not play such an important role in their innate immune responses, and this affects the downstream responses they elicit (e.g. the lack of cathelicidin peptides).^73^ As in humans, DAMPs such as HMGB1 have been shown to be elevated in rats following traumatic injury; however like mice, rats display reduced sensitivity to LPS than humans.^74^ This may be related to the fact that the extracellular domain of rat TLR4 only shares 61% homology with that of human TLR4. Furthermore, there are some subtle differences in PRR receptor expression in comparison with humans. For example, rat dendritic cells express TLR4, whereas human dendritic cells do not,^74^ and whilst human lungs express modest levels TLR4, rat lungs predominantly express TLR.^75^ Nevertheless, rat responses have been widely reported to recapitulate many inflammatory and physiological processes observed in humans including DAMP release, pro‐inflammatory cytokine release, endothelial glycocalyx degradation and altered transcriptional profiles of inflammatory markers.^76^, ^77^, ^78^ Additionally, lymphopenia and increased neutrophil count are recapitulated in rat models alongside decreased NK cell cytotoxicity following trauma.^79^

### Rabbit

Despite not being a member of the rodent family, the immune system of a rabbit is remarkably similar to both the rat and mice.^62^ Rabbits possess a higher (>30%) neutrophil fraction than rodents but have a comparably higher lymphocyte percentage, which is more similar to rodents than humans (60%–70%).^80^ Despite this, the rabbit gene orthologs are more similar to humans than rodents.^81^ Their ease of handling and breeding, accessibility and relative lack of expense means they are commonly used in scientific experimentation.^82^ Typically, rabbits are more widely ethically accepted than larger animal models such as non‐human primates, but boast an increased size and TBV when compared to rodents.

The innate ability of rabbits to produce 13 classes of IgA antibodies is distinct from most other mammals including humans.^82^ Despite most commonly being utilized for their antibody repertoire, rabbit models have many advantages for use in studying traumatic injury. The similarities between rabbit and humans in many chemokine ligand and receptor interactions (e.g. CCR5) and metabolism (e.g. cytochrome P450) facilitate robust investigations into many aspects of inflammation and drug metabolism.^82^ TLR4 expression is broadly similar in rabbits compared to humans, although unlike humans where TLR4 levels are highest in the spleen, the highest levels of TLR4 are found in the lungs and bone marrow.^74^ In contrast to rodents, rabbits display similar sensitivity to LPS as humans.^74^ Additionally, rabbits display comparable neutrophil responses observed following trauma, including their ability to produce ROS.^81^

Although rabbit bones are dissimilar to human in microstructure, skeletal growth and vascular tissue, they may create a more robust model for bone fracture than rodents in many ways. Their size, similarity in bone mineral density and mid‐diaphyseal thickness provide invaluable tools when investigating traumatic bone injury.^83^ Unlike rodents, the size of rabbit models allows more physiological investigations into complex traumas including blast injury that have demonstrated a remarkably similar response to that reported in human patients. This includes the induction of systemic inflammatory response with rapid release of pro‐inflammatory cytokines.^82^

Rabbit models of tissue injury and haemorrhage are able to reproduce the shock responses observed in humans, with a greater base deficit, compensatory hyperventilation and increased mortality.^84^ Similarly, these insults damage the endothelium, increase oxidative stress and induce the release of pro‐inflammatory cytokines.^85^, ^86^

### Pig

The domestic pig is an essential preclinical model; it has a greater clinical relevance than the other species described due to their genetic, physiological and anatomical similarities to humans.^87^ Despite being a large animal model, they are still more ethically accepted than non‐human primates, but less preferable than small animal models. Not only do they have the capacity to almost completely reproduce human immune responses, they also have comparable lymphoid structures, wound healing mechanisms and immunological niches (e.g. palatine and nasopharyngeal tonsils).^88^ Ultimately, porcine models bring great advantages for studying immune interactions and the mechanistic responses to trauma.

Pigs possess many of the same PRRs and downstream signalling pathways as humans. Porcine TLR1‐10 and Nod‐like receptors (NLR)‐1 and NLR2 share over 80% homology with their human gene counterparts, including their ligands and downstream signalling pathways (e.g. NF‐κB, IRF‐3‐7). Therefore, porcine innate immune responses are largely comparable to that of humans and provide an invaluable study tool.^87^ Pigs have been used to model fractures and bone repair, blunt injury, blast injury, shock, TBI and fluid resuscitation strategies.^47^, ^89^ Many of the inflammatory mediators measured in these models recapitulate those observed in humans, including elevated TNFα, IL‐10 and IL‐6.^31^ Similarly, marked increases in DAMPs have also been reported in porcine trauma models, including HMGB1, genomic DNA, ATP and extracellular histones.^90^ As in humans, HMGB1 is elevated early after injury (<2.5 h), correlating with haemorrhage severity, suggesting that hypoperfusion contributes towards tissue damage and HMGB1 release.^90^ As in rodents, increased neutrophil and endothelial cell activation has been reported in porcine models of haemorrhagic shock.^91^ Similarly, increased numbers of alveolar macrophages have been observed in lavage fluid post‐trauma (e.g. blunt chest trauma).^59^ Non‐blood‐derived fluid resuscitation methods in uncontrolled haemorrhage reportedly have no effect on the inflammatory response induced by trauma, with comparable levels of IL‐6, TNFα and G‐CSF reported in pigs treated with either lactated Ringer's solution or normal saline.^92^

Nevertheless, there are still many factors that make the porcine immune system distinct from humans. Despite similarities in lymphocyte education processes and locations, there is a significant fraction (up to 64%) of T cells emerging from thymic tissues as CD4^+^CD8^+^ double‐positive T cells that develop into a memory T‐cell subset with high antigen recall.^93^ Healthy humans, however, only possess very low percentages (<3%) of CD4^+^CD8^+^ T cells in peripheral circulation. Furthermore, pigs have a higher percentage (~30%) of γδ T cells than humans (<10%).^94^ Overall, pig immune cell composition remains more similar to rodents than humans, with 30%–70% lymphocytes and 20%–45% neutrophils.^95^ Similarly, porcine immune systems possess a much higher percentage of both γδ T cells (>15%) and NK cells (3%–5%) than human immune systems. Yet, porcine immune responses have been demonstrated to recapitulate 80% of the responses measured in humans (e.g. trauma‐induced T‐cell lymphopenia), which undoubtedly improves the quality of scientific data acquired from these models.^87^ Pigs have a contractile spleen that can sequester over 20% of red blood cells, and as such, a number of researchers perform a splenectomy to remove the effect of this reflex response.^96^, ^97^ This is of particular importance where there may be difference in the sympathetic response to injury between groups, as occurs following blast injury.^98^ However, as splenectomies are invasive procedures that would result in the initiation of immune responses prior to the experimental injury, careful consideration must be applied in the context of the research question at hand.

Due to their size and requirements, porcine models require much more complex medical equipment, are more challenging to house and can be more expensive than other animal models. Additionally, despite the high level of porcine to human cross‐reactivity, it is still challenging to identify suitable reagents for investigating immune parameters.

### Additional models

Although porcine models are the most common large animal model utilized in trauma research, other species are also used, for example dogs, sheep and NHPs.^99^ These large animal models boast increased TBV and sample sizes, but have more ethical implications and reagents can often be challenging to find. Both ovine and canine models have been used to study TBI and haemorrhagic shock.^100^ A number of relevant haemorrhage models have been developed in dogs; however, their haemodynamic responses to shock may be different to humans, and it is not known whether their immune response(s) to trauma are consistent to that of critically injured human patients.^48^ Furthermore, relatively few reagents are commercially available for studying these immune responses, and as dogs are genetically distinct from humans, using human reagents may not be possible. Due to their similarities to humans, both sheep and goats are good models for bone fractures and healing.^101^ Primate models boast superior hemodynamic and immune responses to many other animal models and have been previously used in models of traumatic injury (e.g. haemorrhagic shock, blunt trauma); however, due to ethical limitations, they are less frequently used today than alternative models.^99^

## OTHER CONSIDERATIONS

Given the varied physiological properties of each species, there are some other important considerations when choosing the correct model. Due to the nature of the studies, these models normally necessitate administration of anaesthesia and may require invasive surgical procedures and/or the use of varied instrumentation prior to the experimental injuries.^102^ These procedures are likely to initiate inflammatory responses themselves, which may have implications on the subsequent immune response to the injury. It is therefore important that consideration is given to the appropriate experimental controls, such as sampling before and after instrumentation/surgery, and the inclusion of sham (uninjured) control groups. Larger animal models allow for greater sample volume (e.g. tissue biopsies and blood volume) and repeated blood sampling over a range of time‐points due to their larger TBV. This creates more freedom to investigate early time‐points, more longitudinal samples or increased downstream analyses. Many studies exploring immune responses to haemorrhage and trauma in preclinical models will include a period of fluid resuscitation in the model; however, there are often differences between studies in the volume and the type of fluid administered. For example, some studies will use autologous blood that has been taken as part of the haemorrhage, whilst others use fluids such as component blood products such as fresh‐frozen plasma (FFP) or crystalloids such as normal saline. Blood products such as FFP have been shown to be beneficial to the endothelium,^77^ but the effects on the inflammatory response are less well defined. Therefore, it is important to consider the exact methods, volumes and timings of infusion and fluid chosen when evaluating models of haemorrhage in order to translate this research into humans and generate clinically relevant data set.^103^ That said, human trauma is very heterogeneous in nature, including in the fluid resuscitation treatments administered (e.g. the type, the volume and timing), and thus, no preclinical model will be 100% representative. Many preclinical studies will use human blood products as resuscitation fluid; however, often little consideration is given to the potential implications of this. For example, human blood products have been shown to transiently suppress mean arterial blood pressure in mice, in which the authors attributed to a xenogeneic reaction.^56^ This reaction to the human products is also likely to elicit an immune reaction, complicating interpretation of results relating to inflammatory changes, and so this must be taken into account when conducting studies of this nature. Furthermore, some of the potential beneficial actions of these products may be missed due to the lack of cross‐reactivity between species. Studies that use resuscitation fluids derived from the same species as the experimental model are much more likely to recapitulate responses seen in humans; however, care must be taken to ensure that the blood processing procedure replicates how they are produced for human clinical use as much as possible.

Most preclinical animal models of complex traumatic injury including haemorrhagic shock are acute in nature (<8 h due to the ethical considerations surround recovery models of severe trauma), yet the development of sepsis and MODS in human patients occurs many days and/or weeks after the initial injury. Extrapolating data from the early phases of trauma may not be representative of changes that occur later down the line, and studies are needed to evaluate whether accurate predictions can be made from acute studies. Studies in mice^104^ and pigs^105^ have extended the experimental protocol up to 72 h, which has highlighted the transient immune responses that occur over this period. For example in a murine polytrauma model, plasma cytokines were elevated the day after injury but returned to baseline levels after 3 days, whereas neutrophilia was evident at both time‐points.^104^ Repeated sampling in a porcine model of polytrauma highlighted IL‐6 was elevated as early as 90 min after injury, peaking after 5.5 h and remaining elevated for 2 days.^105^ These findings highlight the important of longer term studies, especially in models such as the pig that allow for repeated sampling over time. Models of this nature may provide a greater insight into the immune responses that lead to the development of MODS and sepsis following severe injury. That said, immune and endothelial disturbances are seen very early in human patients,^12^, ^28^, ^35^ changes that offer signposts to complications that arise later such as MODS, and so acute preclinical models are representative of the human response to injury for this period. Understanding the early responses, and how they can be modified by therapeutic strategies, is likely to be important in appreciating how these changes may influence the development of post‐trauma complications.

## CONCLUSIONS

Our understanding of immune responses to traumatic injury has rapidly increased in the last decade, which is in no small part due to the use of preclinical animal models. As a result, a number of promising therapeutic strategies that target the immune system are currently being investigated.^106^ In order to ensure the data generated is relevant to the immune responses seen in critically injured human patients, an appreciation of each species strengths and weaknesses is vital to ensure the correct model is chosen for the given research question. The intention of this review is to provide a useful summary of those strengths and weaknesses in the most commonly used species for traumatic haemorrhagic shock research.

## CONFLICT OF INTERESTS

The authors declare no competing interests.

## AUTHOR CONTRIBUTION

J.K.S and R.P made an equal contribution to the writing of this review article. J.K.S and R.P both wrote the first draft, reviewed it and made revisions. Both authors agree on the content.

## Data Availability

Data sharing was not applicable to this article as no data sets were generated or analysed during the current study.

## References

[1] WHO . Disease burden and mortality estimates, CAUSE‐SPECIFIC MORTALITY, 2000–2016 [Internet]. [cited 28/04/2020].

[2] Frohlich M , Lefering R , Probst C , Paffrath T , Schneider MM , Maegele M , et al. Epidemiology and risk factors of multiple‐organ failure after multiple trauma: an analysis of 31,154 patients from the TraumaRegister DGU. J Trauma Acute Care Surg. 2014;76(4):921‐8; discussion 7–8. 24662853 10.1097/TA.0000000000000199

[3] Penn‐Barwell JG , Roberts SAG , Midwinter MJ , Bishop JRB . Improved survival in UK combat casualties from Iraq and Afghanistan. J Trauma Acute Care Surg. 2017;78(5):2003‐12. 10.1097/TA.0000000000000580 25909424

[4] Wafaisade A , Lefering R , Bouillon B , Sakka SG , Thamm OC , Paffrath T , et al. Epidemiology and risk factors of sepsis after multiple trauma: an analysis of 29,829 patients from the Trauma Registry of the German Society for Trauma Surgery. Crit Care Med. 2011;39(4):621‐8. 21242798 10.1097/CCM.0b013e318206d3df

[5] Sauaia A , Moore EE , Johnson JL , Chin TL , Banerjee A , Sperry JL , et al. Temporal trends of postinjury multiple‐organ failure: still resource intensive, morbid, and lethal. J Trauma Acute Care Surg. 2014;76(3):582‐93. 24553523 10.1097/TA.0000000000000147 PMC4116088

[6] Osuka A , Ogura H , Ueyama M , Shimazu T , Lederer JA . Immune response to traumatic injury: harmony and discordance of immune system homeostasis. Acute Med Surg. 2014;1(2):63‐9. 29930824 10.1002/ams2.17 PMC5997205

[7] Lord JM , Midwinter MJ , Chen Y‐F , Belli A , Brohi K , Kovacs EJ , et al. The systemic immune response to trauma: an overview of pathophysiology and treatment. Lancet 2014;384(9952):1455‐65. 25390327 10.1016/S0140-6736(14)60687-5 PMC4729362

[8] Zhang Q , Raoof M , Chen Y , Sumi Y , Sursal T , Junger W , et al. Circulating mitochondrial DAMPs cause inflammatory responses to injury. Nature 2010;464(7285):104‐7. 20203610 10.1038/nature08780 PMC2843437

[9] Kim SY , Son M , Lee SE , Park IH , Kwak MS , Han M , et al. High‐mobility group box 1‐induced complement activation causes sterile inflammation. Front Immunol. 2018;9:705. 29696019 10.3389/fimmu.2018.00705 PMC5904255

[10] Jastrow KM , Gonzalez EA , McGuire MF , Suliburk JW , Kozar RA , Iyengar S , et al. Early cytokine production risk stratifies trauma patients for multiple organ failure. J Am Coll Surg. 2009;209(3):320‐31. 19717036 10.1016/j.jamcollsurg.2009.05.002

[11] Xiao W , Mindrinos MN , Seok J , Cuschieri J , Cuenca AG , Gao H , et al. A genomic storm in critically injured humans. J Exp Med. 2011;208(13):2581‐90. 22110166 10.1084/jem.20111354 PMC3244029

[12] Hazeldine J , Naumann DN , Toman E , Davies D , Bishop JRB , Su Z , et al. Prehospital immune responses and development of multiple organ dysfunction syndrome following traumatic injury: a prospective cohort study. PLoS Medicine 2017;14(7):e1002338. 28719602 10.1371/journal.pmed.1002338 PMC5515405

[13] Timmermans K , Kox M , Vaneker M , van den Berg M , John A , van Laarhoven A , et al. Plasma levels of danger‐associated molecular patterns are associated with immune suppression in trauma patients. Intensive Care Med. 2016;42(4):551‐61. 26912315 10.1007/s00134-015-4205-3 PMC5413532

[14] Andersson U , Wang H , Palmblad K , Aveberger AC , Bloom O , Erlandsson‐Harris H , et al. High mobility group 1 protein (HMG‐1) stimulates proinflammatory cytokine synthesis in human monocytes. J Exp Med. 2000;192(4):565‐70. 10952726 10.1084/jem.192.4.565 PMC2193240

[15] Relja B , Land WG . Damage‐associated molecular patterns in trauma. Eur J Trauma Emerg Surg 2020;46(4):751‐75. 31612270 10.1007/s00068-019-01235-w PMC7427761

[16] Vourc’h M , Roquilly A , Asehnoune K . Trauma‐induced damage‐associated molecular patterns‐mediated remote organ injury and immunosuppression in the acutely ill patient. Front Immunol. 2018;9:1330. 29963048 10.3389/fimmu.2018.01330 PMC6013556

[17] Leijte GP , Custers H , Gerretsen J , Heijne A , Roth J , Vogl T , et al. Increased plasma levels of danger‐associated molecular patterns are associated with immune suppression and postoperative infections in patients undergoing cytoreductive surgery and hyperthermic intraperitoneal chemotherapy. Front Immunol. 2018;9:663.29675023

[18] Cohen MJ , Brohi K , Calfee CS , Rahn P , Chesebro BB , Christiaans SC , et al. Early release of high mobility group box nuclear protein 1 after severe trauma in humans: role of injury severity and tissue hypoperfusion. Crit Care. 2009;13(6):R174. 19887013 10.1186/cc8152 PMC2811903

[19] Stortz JA , Hawkins RB , Holden DC , Raymond SL , Wang Z , Brakenridge SC , et al. Cell‐free nuclear, but not mitochondrial, DNA concentrations correlate with the early host inflammatory response after severe trauma. Sci Rep. 2019;9(1):13648. 31541163 10.1038/s41598-019-50044-z PMC6754448

[20] Wang X‐W , Karki A , Zhao X‐J , Xiang X‐Y , Lu Z‐Q . High plasma levels of high mobility group box 1 is associated with the risk of sepsis in severe blunt chest trauma patients: a prospective cohort study. J Cardiothorac Surg. 2014;9(1):133. 25085006 10.1186/s13019-014-0133-5 PMC4132233

[21] Fan J , Li Y , Levy RM , Fan JJ , Hackam DJ , Vodovotz Y , et al. Hemorrhagic shock induces NAD(P)H oxidase activation in neutrophils: role of HMGB1‐TLR4 signaling. J Immunol. 2007;178(10):6573‐80. 17475888 10.4049/jimmunol.178.10.6573

[22] Orlova VV , Choi EY , Xie C , Chavakis E , Bierhaus A , Ihanus E , et al. A novel pathway of HMGB1‐mediated inflammatory cell recruitment that requires Mac‐1‐integrin. EMBO J. 2007;26(4):1129‐39. 17268551 10.1038/sj.emboj.7601552 PMC1852832

[23] Treutiger CJ , Mullins GE , Johansson A‐SM , Rouhiainen A , Rauvala HME , Erlandsson‐Harris H , et al. High mobility group 1 B‐box mediates activation of human endothelium. J Intern Med. 2003;254(4):375‐85. 12974876 10.1046/j.1365-2796.2003.01204.x

[24] Wolfson RK , Chiang ET , Garcia JGN . HMGB1 induces human lung endothelial cell cytoskeletal rearrangement and barrier disruption. Microvasc Res. 2011;81(2):189‐97. 21146549 10.1016/j.mvr.2010.11.010 PMC3678727

[25] Johansson PI , Stensballe J , Rasmussen LS , Ostrowski SR . A high admission syndecan‐1 level, a marker of endothelial glycocalyx degradation, is associated with inflammation, protein C depletion, fibrinolysis, and increased mortality in trauma patients. Ann Surg. 2011;254(2):194‐200. 21772125 10.1097/SLA.0b013e318226113d

[26] Gonzalez Rodriguez E , Ostrowski SR , Cardenas JC , Baer LA , Tomasek JS , Henriksen HH , et al. Syndecan‐1: a quantitative marker for the endotheliopathy of trauma. J Am Coll Surg. 2017;225(3):419‐27. 28579548 10.1016/j.jamcollsurg.2017.05.012

[27] Ostrowski SR , Henriksen HH , Stensballe J , Gybel‐Brask M , Cardenas JC , Baer LA , et al. Sympathoadrenal activation and endotheliopathy are drivers of hypocoagulability and hyperfibrinolysis in trauma: A prospective observational study of 404 severely injured patients. J Trauma Acute Care Surg. 2017;82(2):293‐301. 27779595 10.1097/TA.0000000000001304

[28] Naumann DN , Hazeldine J , Davies DJ , Bishop J , Midwinter MJ , Belli A , et al. Endotheliopathy of trauma is an on‐scene phenomenon, and is associated with multiple organ dysfunction syndrome: a prospective observational study. Shock. 2018;49(4):420‐8. 28945676 10.1097/SHK.0000000000000999

[29] Scheibner KA , Lutz MA , Boodoo S , Fenton MJ , Powell JD , Horton MR . Hyaluronan fragments act as an endogenous danger signal by engaging TLR2. J Immunol. 2006;177(2):1272‐81. 16818787 10.4049/jimmunol.177.2.1272

[30] Goodall KJ , Poon IKH , Phipps S , Hulett MD . Soluble heparan sulfate fragments generated by heparanase trigger the release of pro‐inflammatory cytokines through TLR‐4. PLoS One 2014;9(10):e109596. 25295599 10.1371/journal.pone.0109596 PMC4190175

[31] Baker TA , Romero J , Bach HH , Strom JA , Gamelli RL , Majetschak M . Systemic release of cytokines and heat shock proteins in porcine models of polytrauma and hemorrhage. Crit Care Med. 2012;40(3):876‐85. 21983369 10.1097/CCM.0b013e318232e314 PMC3383056

[32] Manson J , Cole E , De'Ath HD , Vulliamy P , Meier U , Pennington D , et al. Early changes within the lymphocyte population are associated with the development of multiple organ dysfunction syndrome in trauma patients. Crit Care. 2016;20(1):176. 27268230 10.1186/s13054-016-1341-2 PMC4895987

[33] Bogner V , Keil L , Kanz KG , Kirchhoff C , Leidel BA , Mutschler W , et al. Very early posttraumatic serum alterations are significantly associated to initial massive RBC substitution, injury severity, multiple organ failure and adverse clinical outcome in multiple injured patients. Eur J Med Res. 2009;14(7):284‐91. 19661010 10.1186/2047-783X-14-7-284 PMC3458638

[34] Summers C , Rankin SM , Condliffe AM , Singh N , Peters AM , Chilvers ER . Neutrophil kinetics in health and disease. Trends Immunol. 2010;31(8):318‐24. 20620114 10.1016/j.it.2010.05.006 PMC2930213

[35] Cabrera CP , Manson J , Shepherd JM , Torrance HD , Watson D , Longhi MP , et al. Signatures of inflammation and impending multiple organ dysfunction in the hyperacute phase of trauma: a prospective cohort study. PLoS Medicine 2017;14(7):e1002352. 28715416 10.1371/journal.pmed.1002352 PMC5513400

[36] Hazeldine J , Dinsdale RJ , Harrison P , Lord JM . Traumatic injury and exposure to mitochondrial‐derived damage associated molecular patterns suppresses neutrophil extracellular trap formation. Front Immunol. 2019;10:685. 31001279 10.3389/fimmu.2019.00685 PMC6455291

[37] Hazeldine J , Hampson P , Lord JM . The impact of trauma on neutrophil function. Injury 2014;45(12):1824‐33. 25106876 10.1016/j.injury.2014.06.021

[38] Liao Y , Liu P , Guo F , Zhang Z‐Y , Zhang Z . Oxidative burst of circulating neutrophils following traumatic brain injury in human. PLoS One 2013;8(7):e68963. 23894384 10.1371/journal.pone.0068963 PMC3722225

[39] Paunel‐Görgülü A , Zörnig M , Lögters T , Altrichter J , Rabenhorst U , Cinatl J , et al. Mcl‐1‐mediated impairment of the intrinsic apoptosis pathway in circulating neutrophils from critically ill patients can be overcome by fas stimulation. J Immunol. 2009;183(10):6198‐206. 19841168 10.4049/jimmunol.0901264

[40] Pillay J , Kamp VM , van Hoffen E , Visser T , Tak T , Lammers J‐W , et al. A subset of neutrophils in human systemic inflammation inhibits T cell responses through Mac‐1. J Clin Invest. 2012;122(1):327‐36. 22156198 10.1172/JCI57990 PMC3248287

[41] Heffernan DS , Monaghan SF , Thakkar RK , Machan JT , Cioffi WG , Ayala A . Failure to normalize lymphopenia following trauma is associated with increased mortality, independent of the leukocytosis pattern. Crit Care. 2012;16(1):R12. 22264310 10.1186/cc11157 PMC3396248

[42] Gouel‐Chéron A , Venet F , Allaouchiche B , Monneret G . CD4+ T‐lymphocyte alterations in trauma patients. Crit Care. 2012;16(3):432. 22734607 10.1186/cc11376 PMC3580654

[43] Tschöp J , Martignoni A , Goetzman HS , Choi LG , Wang Q , Noel JG , et al. Gammadelta T cells mitigate the organ injury and mortality of sepsis. J Leukoc Biol. 2008;83(3):581‐8. 18063696 10.1189/jlb.0707507 PMC2747639

[44] Bandyopadhyay G , De A , Laudanski K , Li F , Lentz C , Bankey P , et al. Negative signaling contributes to T‐cell anergy in trauma patients. Crit Care Med. 2007;35(3):794‐801. 17255857 10.1097/01.CCM.0000256847.61085.A5

[45] Cole E , Gillespie S , Vulliamy P , Brohi K , Organ Dysfunction in Trauma Study c . Multiple organ dysfunction after trauma. Br J Surg. 2020;107(4):402‐12. 31691956 10.1002/bjs.11361 PMC7078999

[46] Chang R , Holcomb JB . Optimal fluid therapy for traumatic hemorrhagic shock. Crit Care Clin. 2017;33(1):15‐36. 27894494 10.1016/j.ccc.2016.08.007 PMC5131713

[47] Frink M , Andruszkow H , Zeckey C , Krettek C , Hildebrand F . Experimental trauma models: an update. J Biomed Biotechnol. 2011;2011:797383. 21331361 10.1155/2011/797383 PMC3035380

[48] Hauser CJ . Preclinical models of traumatic, hemorrhagic shock. Shock. 2005;24(Suppl 1):24‐32. 16374369 10.1097/01.shk.0000191387.18818.43

[49] Zschaler J , Schlorke D , Arnhold J . Differences in innate immune response between man and mouse. Crit Rev Immunol. 2014;34(5):433‐54. 25404048

[50] Bryant CE , Monie TP . Mice, men and the relatives: cross‐species studies underpin innate immunity. Open Biol. 2012;2(4):120015. 22724060 10.1098/rsob.120015 PMC3376732

[51] Haley PJ . Species differences in the structure and function of the immune system. Toxicology 2003;188(1):49‐71. 12748041 10.1016/s0300-483x(03)00043-x

[52] Manson J , Hoffman R , Chen S , Ramadan MH , Billiar TR . Innate‐like lymphocytes are immediate participants in the hyper‐acute immune response to trauma and hemorrhagic shock. Front Immunol. 2019;10:1501. 31354702 10.3389/fimmu.2019.01501 PMC6638190

[53] Michel T , Poli A , Domingues O , Mauffray M , Theresine M , Brons NH , et al. Mouse lung and spleen natural killer cells have phenotypic and functional differences, in part influenced by macrophages. PLoS One 2012;7(12):e51230. 23227255 10.1371/journal.pone.0051230 PMC3515449

[54] Mestas J , Hughes CC . Of mice and not men: differences between mouse and human immunology. J Immunol. 2004;172(5):2731‐8. 14978070 10.4049/jimmunol.172.5.2731

[55] Kawasaki T , Suzuki T , Choudhry MA , Bland KI , Chaudry IH . Salutary effects of 17beta‐estradiol on Peyer's patch T cell functions following trauma‐hemorrhage. Cytokine 2010;51(2):166‐72. 20400328 10.1016/j.cyto.2010.03.016 PMC2900535

[56] Potter DR , Baimukanova G , Keating SM , Deng X , Chu JA , Gibb SL , et al. Fresh frozen plasma and spray‐dried plasma mitigate pulmonary vascular permeability and inflammation in hemorrhagic shock. J Trauma Acute Care Surg. 2015;78(6 Suppl 1):S7‐s17. 26002267 10.1097/TA.0000000000000630

[57] Kawasaki T , Fujimi S , Lederer JA , Hubbard WJ , Choudhry MA , Schwacha MG , et al. Trauma‐hemorrhage induces depressed splenic dendritic cell functions in mice. J Immunol. 2006;177(7):4514‐20. 16982888 10.4049/jimmunol.177.7.4514

[58] Strong VE , Mackrell PJ , Concannon EM , Naama HA , Schaefer PA , Shaftan GW , et al. Blocking prostaglandin E2 after trauma attenuates pro‐inflammatory cytokines and improves survival. Shock. 2000;14(3):374‐9. 11028559 10.1097/00024382-200014030-00023

[59] Niesler U , Palmer A , Radermacher P , Huber‐Lang MS . Role of alveolar macrophages in the inflammatory response after trauma. Shock. 2014;42(1):3‐10. 24667621 10.1097/SHK.0000000000000167

[60] Cao Y , Gao Y , Xu S , Bao J , Lin Y , Luo X , et al. Glutamate carboxypeptidase II gene knockout attenuates oxidative stress and cortical apoptosis after traumatic brain injury. BMC Neurosci. 2016;17:15. 27091009 10.1186/s12868-016-0251-1 PMC4836105

[61] Seok J , Warren HS , Cuenca AG , Mindrinos MN , Baker HV , Xu W , et al. Genomic responses in mouse models poorly mimic human inflammatory diseases. Proc Natl Acad Sci USA. 2013;110(9):3507‐12. 23401516 10.1073/pnas.1222878110 PMC3587220

[62] Mullins LJ , Mullins JJ . Insights from the rat genome sequence. Genome Biol. 2004;5(5):221. 15128437 10.1186/gb-2004-5-5-221 PMC416459

[63] Rew DA . The sequencing of the rat genome. Eur J Surg Oncol. 2004;30(8):905‐6. 15497215 10.1016/j.ejso.2004.05.012

[64] Franch A , Castellote C , Pelegri C , Tolosa E , Castell M , Blood B . T, CD4+ and CD8+ lymphocytes in female Wistar rats. Ann Hematol. 1993;67(3):115‐8. 8104040 10.1007/BF01701732

[65] Becker KJ . Strain‐related differences in the immune response: relevance to human stroke. Transl Stroke Res. 2016;7(4):303‐12. 26860504 10.1007/s12975-016-0455-9 PMC4929040

[66] Yang X , Zhou J , He J , Liu J , Wang H , Liu Y , et al. An immune system‐modified rat model for human stem cell transplantation research. Stem Cell Rep. 2018;11(2):514‐21. 10.1016/j.stemcr.2018.06.004 PMC6092637 29983387

[67] Petratos PB , Chen J , Soslow RA , Bleustein CB , Felsen D , Poppas DP . Full‐thickness human foreskin transplantation onto nude rats as an in vivo model of acute human wound healing. Plast Reconstr Surg. 2003;111(6):1988‐97. 12711961 10.1097/01.PRS.0000056831.87062.4B

[68] Hussar P , Piirsoo A , Martson A , Toom A , Haviko T , Hussar U . Bone healing models in rat tibia after different injuries. Ann Chir Gynaecol. 2001;90(4):271‐9. 11820416

[69] Brown RF , Cooper GJ , Maynard RL . The ultrastructure of rat lung following acute primary blast injury. Int J Exp Pathol. 1993;74(2):151‐62. 8499315 PMC2002117

[70] Jones CN , Hoang AN , Martel JM , Dimisko L , Mikkola A , Inoue Y , et al. Microfluidic assay for precise measurements of mouse, rat, and human neutrophil chemotaxis in whole‐blood droplets. J Leukoc Biol. 2016;100(1):241‐7. 26819316 10.1189/jlb.5TA0715-310RR PMC6608085

[71] Fujiyoshi N , Feketeova E , Lu Q , Xu DZ , Hasko G , Deitch EA . Amiloride moderates increased gut permeability and diminishes mesenteric lymph‐mediated priming of neutrophils in trauma/hemorrhagic shock. Surgery 2006;140(5):810‐7. 17084725 10.1016/j.surg.2006.03.003

[72] Xu P , Wen Z , Shi X , Li Y , Fan L , Xiang M , et al. Hemorrhagic shock augments Nlrp3 inflammasome activation in the lung through impaired pyrin induction. J Immunol. 2013;190(10):5247‐55. 23585683 10.4049/jimmunol.1203182 PMC3646900

[73] Schauber J , Dorschner RA , Coda AB , Buchau AS , Liu PT , Kiken D , et al. Injury enhances TLR2 function and antimicrobial peptide expression through a vitamin D‐dependent mechanism. J Clin Invest. 2007;117(3):803‐11. 17290304 10.1172/JCI30142 PMC1784003

[74] Vaure C , Liu Y . A comparative review of toll‐like receptor 4 expression and functionality in different animal species. Front Immunol 2014;5:316. 25071777 10.3389/fimmu.2014.00316 PMC4090903

[75] Weber B , Lackner I , Haffner‐Luntzer M , Palmer A , Pressmar J , Scharffetter‐Kochanek K , et al. Modeling trauma in rats: similarities to humans and potential pitfalls to consider. J Transl Med. 2019;17(1):305. 31488164 10.1186/s12967-019-2052-7 PMC6728963

[76] Mercken EM , Crosby SD , Lamming DW , JeBailey L , Krzysik‐Walker S , Villareal DT , et al. Calorie restriction in humans inhibits the PI3K/AKT pathway and induces a younger transcription profile. Aging Cell 2013;12(4):645‐51. 23601134 10.1111/acel.12088 PMC3714316

[77] Kozar RA , Peng Z , Zhang R , Holcomb JB , Pati S , Park P , et al. Plasma restoration of endothelial glycocalyx in a rodent model of hemorrhagic shock. Anesth Analg. 2011;112(6):1289‐95. 21346161 10.1213/ANE.0b013e318210385c PMC3102787

[78] Xu L , Zhao K , Shen X , Fan X‐X , Ding K , Liu R‐M , et al. Blockade of extracellular high‐mobility group box 1 attenuates systemic inflammation and coagulation abnormalities in rats with acute traumatic coagulopathy. Med Sci Monit. 2016;22:2561‐70. 27436061 10.12659/MSM.900018 PMC4965062

[79] Hirsh M , Dyugovskaya L , Bashenko Y , Krausz MM . Reduced rate of bacterial translocation and improved variables of natural killer cell and T‐cell activity in rats surviving controlled hemorrhagic shock and treated with hypertonic saline. Crit Care Med. 2002;30(4):861‐7. 11940760 10.1097/00003246-200204000-00025

[80] Semerjyan AB , Sargsyan MA , Arzumanyan HH , Hakobyan LH , Abroyan LO , Semerjyan ZB , et al. Immune cell pathology in rabbit hemorrhagic disease. Vet World. 2019;12(8):1332‐40. 31641316 10.14202/vetworld.2019.1332-1340 PMC6755391

[81] Opdahl H , Benestad HB , Nicolaysen G . Differences and similarities between human and rabbit neutrophil granulocyte responses in vitro: the effects of zymosan‐activated plasma, phorbol myristate acetate and n‐formyl‐methionyl‐leucyl‐phenylalanine. Acta Anaesthesiol Scand. 1987;31(6):491‐8. 3630594 10.1111/j.1399-6576.1987.tb02609.x

[82] Esteves PJ , Abrantes J , Baldauf HM , BenMohamed L , Chen Y , Christensen N , et al. The wide utility of rabbits as models of human diseases. Exp Mol Med. 2018;50(5):1‐10. 10.1038/s12276-018-0094-1 PMC5964082 29789565

[83] Mapara M , Thomas BS , Bhat KM . Rabbit as an animal model for experimental research. Dent Res J. 2012;9(1):111‐8. 10.4103/1735-3327.92960 PMC3283968 22363373

[84] Rezende‐Neto JB , Rizoli SB , Andrade MV , Lisboa TA , Cunha‐Melo JR . Rabbit model of uncontrolled hemorrhagic shock and hypotensive resuscitation. Braz J Med Biol Res. 2010;43(12):1153‐9. 21085888 10.1590/s0100-879x2010007500127

[85] Zhang YM , Gao B , Wang JJ , Sun XD , Liu XW . Effect of Hypotensive resuscitation with a novel combination of fluids in a rabbit model of uncontrolled hemorrhagic shock. PLoS One 2013;8(6):e66916. 23805284 10.1371/journal.pone.0066916 PMC3689663

[86] Spear AM , Davies EM , Taylor C , Whiting R , Macildowie S , Kirkman E , et al. Blast wave exposure to the extremities causes endothelial activation and damage. Shock. 2015;44(5):470‐8. 26418548 10.1097/SHK.0000000000000455 PMC4617286

[87] Mair KH , Sedlak C , Kaser T , Pasternak A , Levast B , Gerner W , et al. The porcine innate immune system: an update. Dev Comp Immunol. 2014;45(2):321‐43. 24709051 10.1016/j.dci.2014.03.022 PMC7103209

[88] Dawson HD , Loveland JE , Pascal G , Gilbert JG , Uenishi H , Mann KM , et al. Structural and functional annotation of the porcine immunome. BMC Genom 2013;14:332. 10.1186/1471-2164-14-332 PMC3658956 23676093

[89] Kirkman E , Watts S . Haemodynamic changes in trauma. Br J Anaesth. 2014;113(2):266‐75. 25038158 10.1093/bja/aeu232

[90] Horst K , Hildebrand F , Pfeifer R , Hübenthal S , Almahmoud K , Sassen M , et al. Impact of haemorrhagic shock intensity on the dynamic of alarmins release in porcine poly‐trauma animal model. Eur J Trauma Emerg Surg. 2016;42(1):67‐75. 26038024 10.1007/s00068-015-0504-1

[91] Senthil M , Brown M , Xu DZ , Lu Q , Feketeova E , Deitch EA . Gut‐lymph hypothesis of systemic inflammatory response syndrome/multiple‐organ dysfunction syndrome: validating studies in a porcine model. J Trauma. 2006;60(5):958‐67; discussion 65–7. 16688055 10.1097/01.ta.0000215500.00018.47

[92] Watters JM , Brundage SI , Todd SR , Zautke NA , Stefater JA , Lam JC , et al. Resuscitation with lactated ringer's does not increase inflammatory response in a Swine model of uncontrolled hemorrhagic shock. Shock. 2004;22(3):283‐7. 15316400 10.1097/01.shk.0000135288.54535.8a

[93] Zuckermann FA . Extrathymic CD4/CD8 double positive T cells. Vet Immunol Immunopathol. 1999;72(1–2):55‐66. 10614493 10.1016/s0165-2427(99)00118-x

[94] Holderness J , Hedges JF , Ramstead A , Jutila MA . Comparative biology of γδ T cell function in humans, mice, and domestic animals. Annu Rev Anim Biosci. 2013;1:99‐124. 25387013 10.1146/annurev-animal-031412-103639

[95] Chevaleyre C , Riou M , Brea D , Vandebrouck C , Barc C , Pezant J , et al. The pig: a relevant model for evaluating the neutrophil serine protease activities during acute pseudomonas aeruginosa lung infection. PLoS One 2016;11(12):e0168577. 27992534 10.1371/journal.pone.0168577 PMC5161375

[96] Watts S , Nordmann G , Brohi K , Midwinter M , Woolley T , Gwyther R , et al. Evaluation of prehospital blood products to attenuate acute coagulopathy of trauma in a model of severe injury and shock in anesthetized pigs. Shock. 2015;44:138‐48. 26177017 10.1097/SHK.0000000000000409 PMC4498650

[97] Jernigan PL , Hoehn RS , Cox D , Heyl J , Dorlac WC , Pritts TA . What if i don't have blood? Hextend is superior to 3% saline in an experimental model of far forward resuscitation after hemorrhage. Shock. 2016;46(3 Suppl 1):148‐53. 27380529 10.1097/SHK.0000000000000676

[98] Sawdon M , Ohnishi M , Watkins PE , Kirkman E . The effects of primary thoracic blast injury and morphine on the response to haemorrhage in the anaesthetised rat. Exp Physiol. 2002;87(6):683‐9. 12447448 10.1113/eph8702432

[99] Tsukamoto T , Pape HC . Animal models for trauma research: what are the options? Shock. 2009;31(1):3‐10. 18636048 10.1097/SHK.0b013e31817fdabf

[100] Vink R . Large animal models of traumatic brain injury. J Neurosci Res. 2018;96(4):527‐35. 28500771 10.1002/jnr.24079

[101] Starr AJ , Welch RD , Eastridge BJ , Pierce W , Zhang H . The effect of hemorrhagic shock in a caprine tibial fracture model. J Orthop Trauma. 2002;16(4):250‐6. 11927806 10.1097/00005131-200204000-00006

[102] Tremoleda JL , Watts SA , Reynolds PS , Thiemermann C , Brohi K . Modeling acute traumatic hemorrhagic shock injury: challenges and guidelines for preclinical studies. Shock. 2017;48(6):610‐23. 28509685 10.1097/SHK.0000000000000901

[103] Hirshberg A , Hoyt DB , Mattox KL . Timing of fluid resuscitation shapes the hemodynamic response to uncontrolled hemorrhage: analysis using dynamic modeling. J Trauma. 2006;60(6):1221‐7. 16766964 10.1097/01.ta.0000220392.36865.fa

[104] Gentile LF , Nacionales DC , Cuenca AG , Armbruster M , Ungaro RF , Abouhamze AS , et al. Identification and description of a novel murine model for polytrauma and shock. Crit Care Med. 2013;41(4):1075‐85. 23399937 10.1097/CCM.0b013e318275d1f9 PMC3666582

[105] Horst K , Simon TP , Pfeifer R , Teuben M , Almahmoud K , Zhi Q , et al. Characterization of blunt chest trauma in a long‐term porcine model of severe multiple trauma. Sci Rep. 2016;6:39659. 28000769 10.1038/srep39659 PMC5175194

[106] Huber‐Lang M , Lambris JD , Ward PA . Innate immune responses to trauma. Nat Immunol. 2018;19(4):327‐41. 29507356 10.1038/s41590-018-0064-8 PMC6027646

